# Identification of QTLs for Rust Resistance in the Peanut Wild Species *Arachis magna* and the Development of KASP Markers for Marker-Assisted Selection

**DOI:** 10.1534/g3.115.018796

**Published:** 2015-05-05

**Authors:** Soraya C. M. Leal-Bertioli, Uiara Cavalcante, Ediene G. Gouvea, Carolina Ballén-Taborda, Kenta Shirasawa, Patrícia M. Guimarães, Scott A. Jackson, David J. Bertioli, Márcio C. Moretzsohn

**Affiliations:** * Embrapa Genetic Resources and Biotechnology, 70770-917 Brasília, DF, Brazil,; † Center for Applied Genetic Technologies, University of Georgia, Athens, Georgia 30602-6810; ‡ University of Brasília, Institute of Biological Sciences, Campus Darcy Ribeiro, 70910-900 Brasília, DF, Brazil; § Kazusa DNA Research Institute, Kisarazu, Chiba, 292-0818, Japan

**Keywords:** *Arachis*, peanut, marker-assisted selection, rust, molecular breeding

## Abstract

Rust is a major pathogen of the peanut crop. Development and adoption of rust-resistant cultivars is the most cost efficient and effective way to control the spread of the disease and reduce yield losses. Some cultivated peanut germplasm accessions have a degree of resistance, but the secondary gene pool is a source of much stronger resistance alleles. Wild species, however, have undesirable agronomic traits that are a disincentive to their use in breeding. The identification of genomic regions that harbor disease resistance in wild species is the first step in the implementation of marker-assisted selection that can speed the introgression of wild disease resistances and the elimination of linkage drag. In this work, we identify genome regions that control different components of rust resistance in a recombinant inbred line population developed from a cross between two *Arachis* species, the susceptible most probable B genome ancestor of cultivated peanut, *Arachis ipaënsis*, and an accession of its closest relative, *Arachis magna*, which is resistant to rust. Quantitative trait loci for several components of resistance were placed in the same position on linkage group B08. Single-nucleotide polymorphism Kompetitive allele-specific polymerase chain reaction markers for rust resistance region were designed and validated for marker function in both diploid and tetraploid contexts.

Peanut or groundnut (*Arachis hypogaea* L.) is a very important oilseed and food crop, cultivated on more than 24 million hectares in tropical and subtropical regions and with a global annual production of approximately 38 million tons (FAOSTAT 2012). It originated in a region encompassing the south of Bolivia and the northwest of Argentina by hybridization of two wild species and spontaneous chromosome duplication to form an allotetraploid.

Peanut is affected by a number of pests and diseases that reduce yield and increase production costs. Rust, a foliar disease caused by *Puccinia arachidis* Speg. is one of the most important and widespread (Subrahmanyam *et al.* 1993). It is believed that the pathosystem rust-peanut co-evolved in Peru, where the host has been cultivated since prehistoric times. The peanut crop spread widely around the world after the Spanish and Portuguese colonization of South America, but the pathogen was largely confined to South America until late 1960s. However, it has since spread to all peanut-growing areas (Hammons 1973; Subrahmanyam *et al.* 1993). The pathogen is host-specific. Losses are particularly severe if the crop is also attacked by leaf spots [*Cercospora arachidicola* Hori and *Cercosporidium personatum* (Berk. and Curt.)]. Unlike leaf spots, rust-infected leaves tend to remain attached to the plant, and this, combined with a short life cycle, favor fast and prolific pathogen multiplication and rapid spread of the disease. Modern locally preferred cultivars generally are susceptible to rust and other foliar diseases. Chemical control of the fungus can be effective but is costly; timing of pesticide application also can be a challenge.

The development and adoption of high-yielding, rust-resistant cultivars is seen as the best strategy to control the spread of the disease (Gibbons 1980). However, no complete resistance has been found in cultivated germplasm (Subrahmanyam *et al.* 1982; Pande and Rao 2001; Fávero *et al.* 2009). This finding is consistent with the recent allotetraploid origin of cultivated peanut and the resulting limited number of disease resistance alleles available in its evolutionary arms race against rust. Additionally, since until late 1960s rust essentially was confined to South America and peanut cultivars elsewhere were not subject to selection pressure. In a wide screen of germplasm, more resistant genotypes were found to be mostly from Peru (Subrahmanyam *et al.* 1989, 1993). Although partially resistant, cultivated peanut genotypes do exist, high levels of resistance or even immunity to rust only are found in the wild species (Pande and Rao 2001; Fávero *et al.* 2009; Leal-Bertioli *et al.* 2010).

Introgression of resistances from diploid wild relatives into allotetraploid peanut involves special crossing schemes to overcome the ploidy differences, followed by multiple rounds of backcrossing (Simpson and Starr 2001; Stalker and Lynch 2002; Simpson *et al.* 2003; Holbrook *et al.* 2008). The use of markers linked to rust resistance are likely to aid in the selection of backcrossed progeny, the breaking of linkage drag, the pyramiding of multiple resitance loci, and in increasing the speed of selection. Previously, a linkage map has been published using a population derived from a cross between the single available accession of the most probable B genome ancestor of peanut, *Arachis ipaënsis* K 30076, and a highly rust-resistant accession of the closely related *Arachis magna*, K 30097 (Moretzsohn *et al.* 2009; Shirasawa *et al.* 2013). Here we report the identification of strong quantitative trait loci (QTL) linked to rust resistance and the development of single-nucleotide polymorphism (SNP) Kompetitive allele-specific polymerase chain reaction (KASP) markers suitable for foreground selection in breeding programs.

## Materials and Methods

### Plant material

The F_6_ population composed of 94 individuals was obtained by single seed descent from the F_2_ population described in Moretzsohn *et al.* (2009). Progenies are derived from a cross between *A. ipaënsis* (accession GKBSPSc 30076, hereafter referred to in the abbreviated form K 30076), and the closely related *A. magna* (GKSSc 30097 hereafter referred to in the abbreviated form K 30097), used as the female and male parents, respectively. Seeds were obtained from the Brazilian *Arachis* germplasm collection, maintained at Embrapa Genetic Resources and Biotechnology (Brasília-DF, Brazil).

### Phenotyping

#### Rust phenotyping:

The recombinant inbred lines (RILs) population and the parents were phenotyped for resistance to *P. arachidis*. *Arachis hypogaea* cv. Runner IAC 886 was included as susceptible control. The population was evaluated on F_6_ and F_7_ generations. Phenotyping was performed using the detached leaf technique (Moraes and Salgado 1982; Leal-Bertioli *et al.* 2009). Field assays would not be suitable because of the architecture of the wild-derived diploid plants. Rust spores were collected from infested peanut plants in Pindorama, São Paulo State, Brazil (coordinates 21.1858° S, 48.9072° W). Two bioassays were done, one in 2012 and the other in 2013. In the bioassay of 2012, leaves were inoculated with ca. 4 × 10^5^ urediniospores/mL in 0.05% Tween 20 fungal spores and maintained at 26–28° and photoperiod of 10-hr light and 14-hr dark. In the bioassay of 2013, ca. 2 × 10^5^ urediniospores/mL were used. Four replicates of each individual were analyzed 25 d after inoculation. Susceptibility was measured using the following parameters: total number of lesions/leaf area (cm) (TL/LA), number of sporulated lesions/leaf area (cm) (SL/LA), Incubation period (time for appearance of first lesion in number of days after inoculation) (IncPer), and susceptibility index (SI). SI was calculated with the scale of Savary *et al.* (1989), with the following modifications: index was the number of lesions times a number that reflected lesion size/reaction. I = ∫(s * n)/LA, where s = lesion size (1 = necrotic aborted lesion, 2−6 = ruptured, sporulating pustules, varying between 0.5 and 3 mm in diameter), n = number of lesions of a particular size, LA = leaf area (mm^2^). Sporulation was evaluated with the aid of a stereoscope microscope. LA was calculated with the software Quant (Vale *et al.* 2001). In genotypes that did not present symptoms and therefore did not have incubation period, for QTL analyses, this trait was artificially tabulated as 200.

#### Other agronomic/domestication traits:

Plants were grown in long trays (1 m × 30 cm × 30 cm), with enough space for lateral branch trailing and seed set. Branches were regularly trailed back to the pots to ensure that pegs would penetrate the soil. At between 40 and 60 d after planting, height of main stem of up to 10 plants of each RIL was measured (main stem height; MSH). At harvest (approximately 120 d after planting), peg length (PL) and pod constriction (PC) was measured with six replications. Harvested seeds were counted (seed number), dried at 20° at 15% relative humidity (RH) for 15 d, and 10 seeds, randomly selected, were weighted (10-SW). Evaluations were performed in three years, 2011, 2012 and 2013, except for PL and PC, which were evaluated only in 2012.

### DNA extraction

Total genomic DNA was extracted from young leaflets essentially as described by Grattapaglia and Sederoff (1994). The quality and quantity of the DNA were evaluated on 1% agarose gel electrophoresis and spectrophotometer NanoDrop 1000 (Thermo Fisher Scientific).

### Genetic mapping and QTL analyses

A linkage map for this population has been published in Shirasawa *et al.* (2013). This map contained 773 microsatellite and 25 miniature inverted-repeat transposable element loci. We used these 798 markers plus 26 newly genotyped microsatellite markers to construct an updated linkage map, by using Mapmaker Macintosh 2.0 and Mapmaker/EXP 3.0 (Lander *et al.* 1987; Lincoln *et al.* 1992). A χ^2^ test was performed to test the null hypothesis of 1:1 segregation on all scored markers. A minimum logarithm of the odds (LOD) score of 6.0 and maximum recombination fraction (θ) of 0.35 were set as thresholds for linkage groups (LGs) determination with the “group” command. The most likely marker order within each LG was estimated by the matrix correlation method using the “first order” command. Marker orders were confirmed by comparing the log-likelihood of the possible orders by permuting all adjacent triple orders (“ripple” command). After establishment of the group orders, the LOD score was set to 3.0 to include additional markers in the groups. The “try” command was then used to determine the exact position of the new markers within each group. The new marker orders were again confirmed with the “ripple” command. Recombination fractions were converted into map distances in centimorgans (cM) using the Kosambi’s mapping function and the “error detection” command available in Mapmaker/EXP 3.0 (Lander *et al.* 1987, Lincoln *et al.* 1992). Based on this map, genomic regions with no recombination or identical markers were identified and all loci but one were removed from these regions (pairs or groups of loci with 0 cM distance).

This newly developed framework map was used for QTL analysis. Phenotyping data included the components of resistance to *P. arachidis* and agronomic traits (Supporting Information, File S1). Traits phenotyped in different trials or bioassays were analyzed separately. The normality of data distribution was evaluated by skewness and kurtosis values using WinQTL Cartographer, version 2.5 (Wang *et al.* 2006). QTL were mapped by using the composite interval mapping method proposed by Zeng (1993, 1994) and also the WinQTL Cartographer. This software assumes that the quantitative data under analysis are normally distributed. Some of the data sets did not fit this assumption and were log(x) transformed. We performed composite interval mapping analysis with the standard model (Model 6), scanning the genetic map and estimating the likelihood of a QTL and its corresponding effects at every 1 cM while using eight significant marker cofactors to adjust the phenotypic effects associated with other positions in the genetic map. A window size of 10 cM was used, and therefore cofactors within 10 cM on either side of the QTL test site were not included in the QTL model. Thresholds were determined for each trait by permutation tests (Churchill and Doerge 1994; Doerge and Churchill 1996), by the use of 1000 permutations and a significance level of 0.05. Graphic presentation of the LGs and the significant QTL was drawn with MapChart, version 2.1 (Voorrips 2002).

### KASP marker development and validation in tetraploid genetics

#### Rationale:

The aim of the methods in this section was to develop reliable and easy-to-use DNA markers for the genomic region in *A. magna* K 30097 that confers rust resistance. Although *A. magna* K 30097 was of primary interest for this study, *A. batizocoi* K9484 also is being used in our research and for introgression in breeding programs (Leal-Bertioli *et al.* 2014b). Therefore, we aimed to develop markers that would function for both these species (Leal-Bertioli *et al.* 2014a,b).

Because introgression will be in allotetraploid cultivated peanut, the markers must function in this genetic context, but for SNP discovery, we used a strategy of SNP calling in the diploid context. This strategy relies on the very close relationship of *A. ipaënsis* and the B genome of *A. hypogaea* (Moretzsohn *et al.* 2013). Because of this close relationship, a polymorphism identified between *A. magna* and *A. ipaënsis* is very likely to be conserved between *A. magna* and the B genome of *A. hypogaea*. After marker design, this conservation was confirmed by marker assays.

#### Production and assembly of transcript sequences:

Total RNA from *A. magna* K 30097 and *A. batizocoi* K9484 was extracted from the first expanded leaf of the main axis using the QIAGEN Plant RNeasy kit (QIAGEN) with on-column DNAse treatment. cDNA libraries were constructed using equal amounts of RNA from five individuals of each genotype using the TruSeq v2 library construction kit (Illumina), as described in Leal-Bertioli *et al.* (2015). To obtain long reads to improve transcriptome assemblies, size-selected libraries were sequenced using MiSEQ v3.0. Adapter and quality trimming was performed using Trim_galore! v0.3.5. (http://www.bioinformatics.babraham.ac.uk/projects/trim_galore/). Adapters were trimmed with Cutadapt (http://code.google.com/p/cutadapt/). FastQC (http://galaxy.csdb.cn:8000/tool_runner?tool_id=fastqc) was used to display quality information for cleaned reads. Transcripts were assembled using Trinity (Haas *et al.* 2013). Assembled transcripts were filtered to include only the longest isoform from each read cluster. The longest isoforms were then aligned to each other by the use of NCBI blastn v2.2.29. Alignments with 100% sequence identity and ≥ 90% sequence length were considered redundant and removed from the final assembly.

#### SNP discovery:

*Arachis ipaënsis* reference genome sequence (www.Peanutbase.org) was used as proxy of the B genome of *A. hypogaea* to discover SNPs between the rust-resistant accessions and peanut (susceptible). This was done by aligning *A. magna* and *A. batizocoi* transcripts (resistant) with the reference genome of *A. ipaënsis* (GenBank assembly accession GCA_000816755.1) using the NGSEP pipeline (Duitama *et al.* 2014) tagging the region where the main QTL for rust resistance was identified (pseudomolecule Araip.B08, peanutbase.org), in the vicinity of the microsatellite marker Ah-280 (region between 117048352 and 129519037 bp) and also for another QTL linked marker on Araip.B08, AHGS1350 (region between 346729 and 848328 bp) (Table 1). Default parameters were used, except the minimum and maximum fragment length for valid paired-end alignments, which we estimated separately for each genotype aligning their first 250,000 fragments and then plotting the distribution of estimated insert lengths (Script available at the NGSEP Web site http://sourceforge.net/projects/ngsep/files/Library/scripts/). We used the recommended parameters of NGSEP for analysis of WGS data: (1) minimum genotype quality 40; (2) minimum value allowed for a base quality score 30; and (3) Maximum number of alignments allowed to start at the same reference site 2. We also used NGSEP for filtering (the most relevant was a maximum minor allele frequency of 0.01) and conversion from VCF to other formats for primer design and visualization of SNPs with Flapjack software (Milne *et al.* 2010).

**Table 1:** Quantitative trait loci identified for resistance to *Puccinia arachidis* and agronomic traits on an *A. ipaënsis* × *A. magna* F_6_ population

Trait Category	Trait Symbol	LG*^a^*	Position*^b^*	Nearest Marker(s)	LOD*^c^*	Additive Effect*^d^*	R^2^ (%)*^e^*
Rust resistance	SI_2012	4	17.0	TC7G10	2.7	0.18	7.8
		7	38.7	AHS0598	3.0	0.16	8.2
		8	25.4	AHGS1350 / AHS2541	3.3	0.20	13.2
		8	35.9	Ah-280	6.9	0.25	21.2
	SI_2013	8	35.9	Ah-280	3.2	0.31	5.8
	TL/LA_2012	8	25.4	AHGS1350 / AHS2541	4.1	0.13	16.0
		8	35.1	Ah-280	2.9	0.09	8.9
	TL/LA_2013	8	35.9	Ah-280	3.8	0.20	12.3
	SL/LA_2012	8	35.9	Ah-280	3.8	0.07	11.1
	SL/LA_2013	8	35.9	Ah-280	3.5	0.14	11.0
	Log_IncPer_2012	8	42.9	Ah-280 / Ah-558	8.2	−0.46	59.3
	Log_IncPer_2013	8	33.1	AHS2541 / Ah-280	7.6	−0.33	33.9
		8	38.9	Ah-280	7.6	−0.33	34.8
Productivity	Log_SN	3	82.3	ML2A05	3.4	−0.11	8.6
		4	28.0	AHGS2785	2.6	−0.10	6.2
		10	35.5	AHS1488	3.1	−0.15	10.3
	10-SW	4	68.8	AHGS1279 / AHS2728	3.9	−0.32	18.4
		5	44.5	AHGS2602	2.9	0.20	8.3
Seed	Peg_Length	1	40.8	AHGS2019 / Seq12B2	3.4	−2.07	7.2
Characteristics		4	64.9	AHGS2155	11.2	4.18	25.8
		4	70.8	AHGS1279 / AHS2728	8.7	4.38	30.2
		5	10.4	AHS2897	3.4	2.06	6.9
		9	43.7	AHGS2018 / AHGS2235	3.0	1.91	6.3
	Pod_constriction	1	37.1	AHGS2332	4.1	−0.67	9.4
		4	64.3	AHGS1917	3.4	0.59	7.4
		5	47.0	AHGS2513	3.5	−0.80	8.0
		6	25.4	AHGS2106	3.7	−0.66	9.7
		8	0.0	AHGS1383	3.5	−0.60	8.1
		9	36.3	AHGS1478_b3 / AHGS2537_b2	3.3	0.56	7.3
Plant	MSH_2009	2	37.8	RN31F06	4.8	−5.44	14.5
Architecture		4	64.3	AHGS1917 / AHGS2155	6.5	−5.79	17.6
		5	48.7	AHGS1228	3.2	3.61	8.1
	Log_MSH2011	4	64.9	AHGS2155	4.1	−0.10	10.7
		5	41.1	AHGS1980	4.0	1.64	10.0
		6	13.0	AHS2153	4.5	−0.11	11.4
	MSH_2012	3	23.2	TC1E06	4.0	−1.97	8.1
		4	64.3	AHGS1917 / AHGS2155	12.3	−3.56	30.4
		5	48.7	AHGS1228	3.6	2.13	7.4

a Linkage group.

b Expressed in Kosambi cM.

c LOD score, logarithm of the odds.

d Positive values indicate that higher-value alleles come from *A. ipaënsis* K 30076, and negative values indicate that higher-value alleles come from *A. magna* K 30097.

e Proportion of the phenotypic variance explained by the quantitative trait loci.

#### Primer design and test:

**Figure 1:**
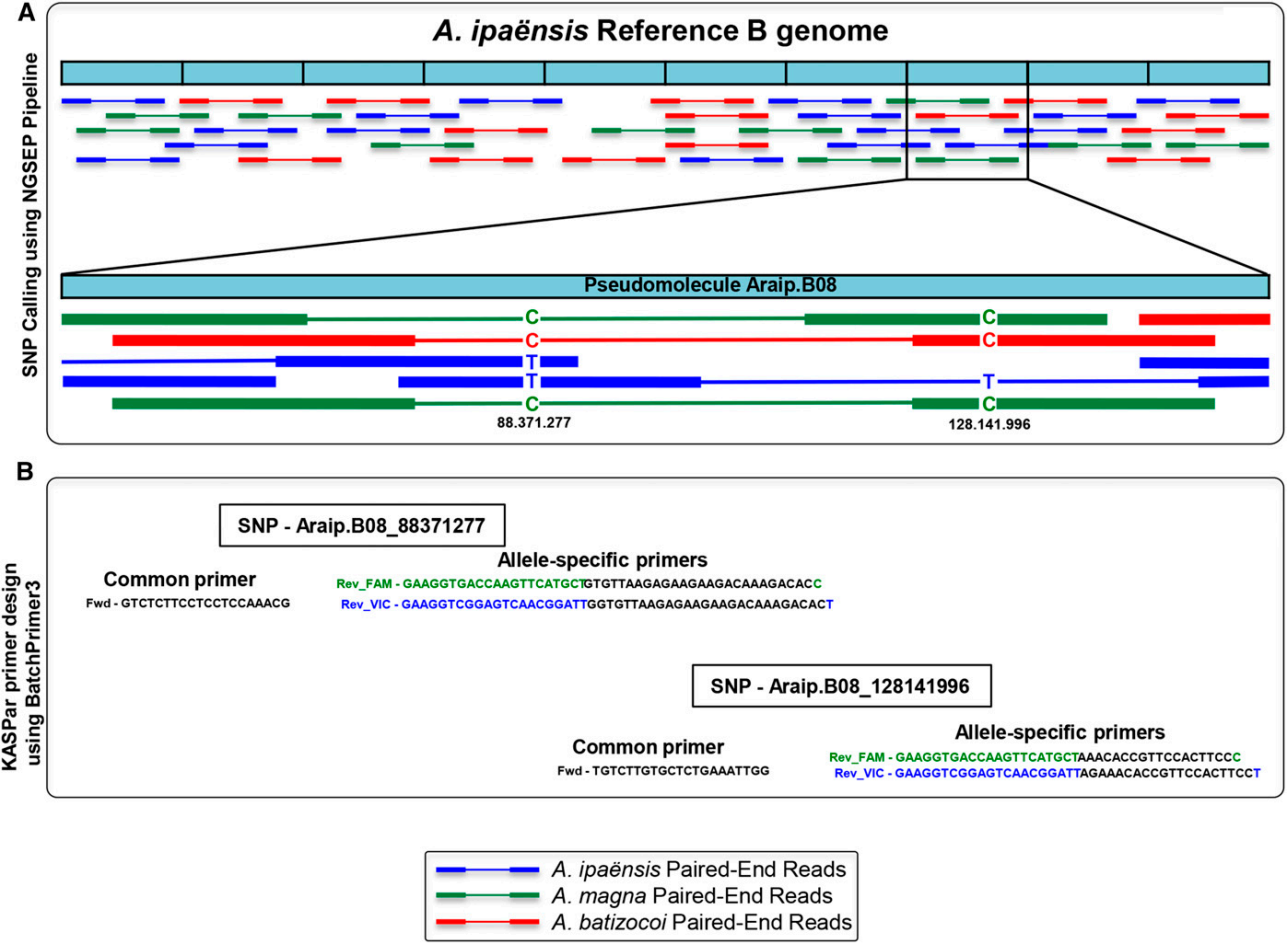
A schematic diagram of single-nucleotide polymorphism (SNP) discovery and Kompetitive allele-specific polymerase chain reaction (KASP) primer design. *A. ipaënsis* K 30076 is used as proxy for the B-genome of *A. hypogaea*. (A) Alignment of *A. ipaënsis* K 30076, *A. magna* K 30097, and *A. batizocoi* K 9484 paired-end cDNA reads onto *A. ipaënsis* K 30076 genomic sequence, Pseudomolecule Araip.B08 (where rust resistance QTL reside) and the identification of SNPs. (B) Example of design of allele-specific and site specific (common) primers for the SNPs identified.

Allele-specific forward primers and a common reverse primer were designed for use in KASP assays (LGC Genomics Ltd. Hoddesdon, UK; http://www.lgcgenomics.com/kasp-genotyping-reagents) using BatchPrimer3 (http://probes.pw.usda.gov/batchprimer3/) with the “Allele specific primers and allele flanking primers” option. The parameters used were 60−120 bp in size, Tm between 58 and 60°, and GC content between 30 and 80%. The alternative alleles were marked with 6-FAM and reference alleles with VIC. For each SNP, two allele-specific forward primers and one common reverse primer were designed. A schematic diagram of SNP discovery and primer design is shown in Figure 1, A and B. Primer information is listed in File S1.

KASP assays were performed with the following genotypes: the diploids *A. ipaënsis* K 30076, *A. batizocoi* K9484 and *A. magna* K 30097, the induced allotetraploids (*A. magna* K 30097 x *A. stenosperma* V15076)^4x^ (here called MagSten) and (*A. batizocoi* K9484 × *A. stenosperma* V10309)^4x^ (here called BatSten1) and six *A. hypogaea* cultivars (Tifrunner, Tifguard, GA-06G, NC3033, ICVG 88145, and SPTG_06). Reactions consisted of 2 μL of KASP 2X reaction mix, 0.055 μL of assay primer mix (12 mM of each allele-specific primer and 30 mM of common primer) and 20 ng of genomic DNA, in a 4-µL volume. A C1000 Thermal Cycler (Bio-Rad) was used with the following cycling conditions: 94° for 15 min, nine cycles of 94° for 20 sec, touchdown starting at 65° for 60 sec (decreasing 0.8° per cycle), 29 cycles of 94° for 20 sec, and 57° for 60 sec (http://www.cerealsdb.uk.net/cerealgenomics/CerealsDB/PDFs/KASP_SNP_Genotyping_Manual.pdf). To improve the results, a second KASP program was run as following: 9 cycles of 94° for 20 sec and 57° for 60 sec. Fluorescence was read by a The LightCycler 480 Instrument II (Roche Life Science) and analyzed using the LightCycler 480 Software (V.1.5.1).

## Results

### Phenotyping

The F_6_ RIL population used here was produced by single seed descent from a cross *A. ipaënsis* × *A. magna*. The F_1_ showed high fertility, reflecting that the parents are very closely related. The population shows large variability for main stem height, length of lateral branches, seed size and number, and resistance to rust. Most individuals show mid-parent values, but the population showed large transgressive segregation. High skewness and kurtosis values showed that four of the 15 traits evaluated were not normally distributed. These were incubation period for 2012 and 2013 (IncPer_2012, IncPer_2013); seed number, average of 3 years; and main stem height for 2011 (MSH_2011). To achieve approximately normal distributions, these four traits were log(x) transformed. All phenotyping information is presented on File S1.

#### Rust:

**Figure 2:**
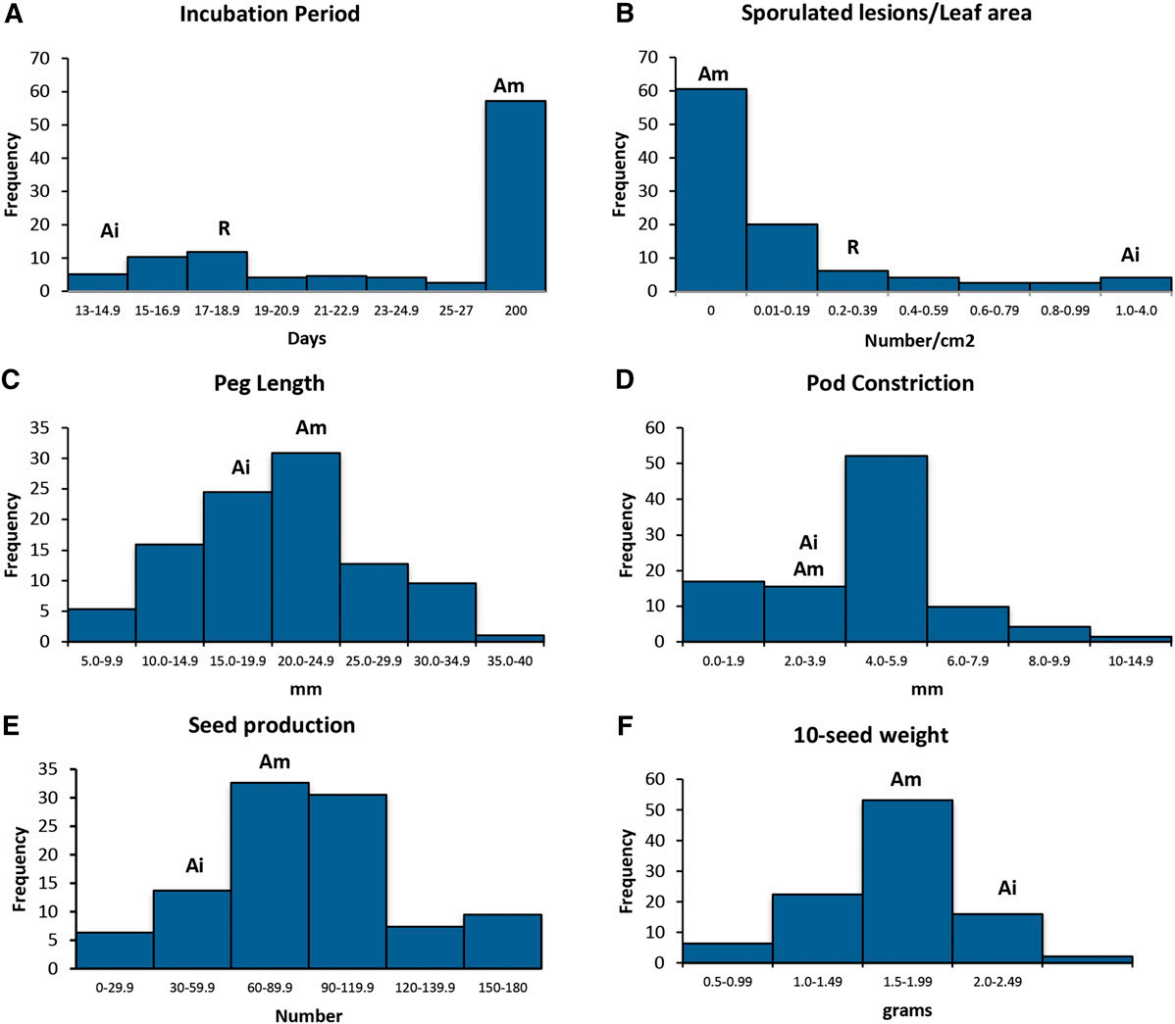
Frequency distribution of rust resistance, peg, and seed characteristics (A−F) in recombinant inbred lines (F_6_/F_7_ RILs) derived from a cross of *A. ipaënsis* K 30076 (Ai) with *A. magna* K 30097 (Am). For rust tests, *A. hypogaea* cv. Runner IAC 886 (R) was the susceptible control. With the exception of pod constriction, for all traits the means of the parents are significantly different (*P* < 0.05). In (A) genotypes without symptoms (therefore without incubation period), this trait was artificially tabulated as 200.

The frequency distribution based on the pooled data for TL/LA, number of SL/LA, and incubation period (IncPer) showed strong biased toward resistance, because 41 individuals and the resistant parent presented no lesions in either experiments (Figure 2, A and B). The susceptible parent *A. ipaënsis* K 30076 showed greater susceptibility than the control, *A. hypogaea* cv. Runner IAC 886, in all components tested: larger SI, TL/LA and SL/LA, and shorter IncPer than the susceptible control (File S1). Seventeen individuals had greater SI, SL/LA, and TL/LA than the susceptible control. In addition, five had shorter IncPer (File S1). As expected, the number and size of lesions were negatively correlated with Incubation period (Pearson r = – 0.6, *P* < 0.005). No chlorosis was observed on any accession of the F_6_/F_7_ population. On leaves of some of the less-susceptible genotypes, necrotic areas corresponding to colonies aborted at a late developmental stage were observed.

#### Other agronomic/domestication traits:

Phenotypic evaluations were performed at different generations. Values were normally distributed for most traits for most years. With the exception of PC and PL, the means of the parents are significantly different (*P* < 0.05). Comparison of the means of the parents and the segregating genotypes reveals that for all traits there is transgressive segregation in the progenies (Figure 2, C−F). On an average for 3 years, *A. magna* produced more seed than *A. ipaënsis*, but 10-seed weight was lower (Figures 2, E and F).

### Map construction

**Figure 3:**
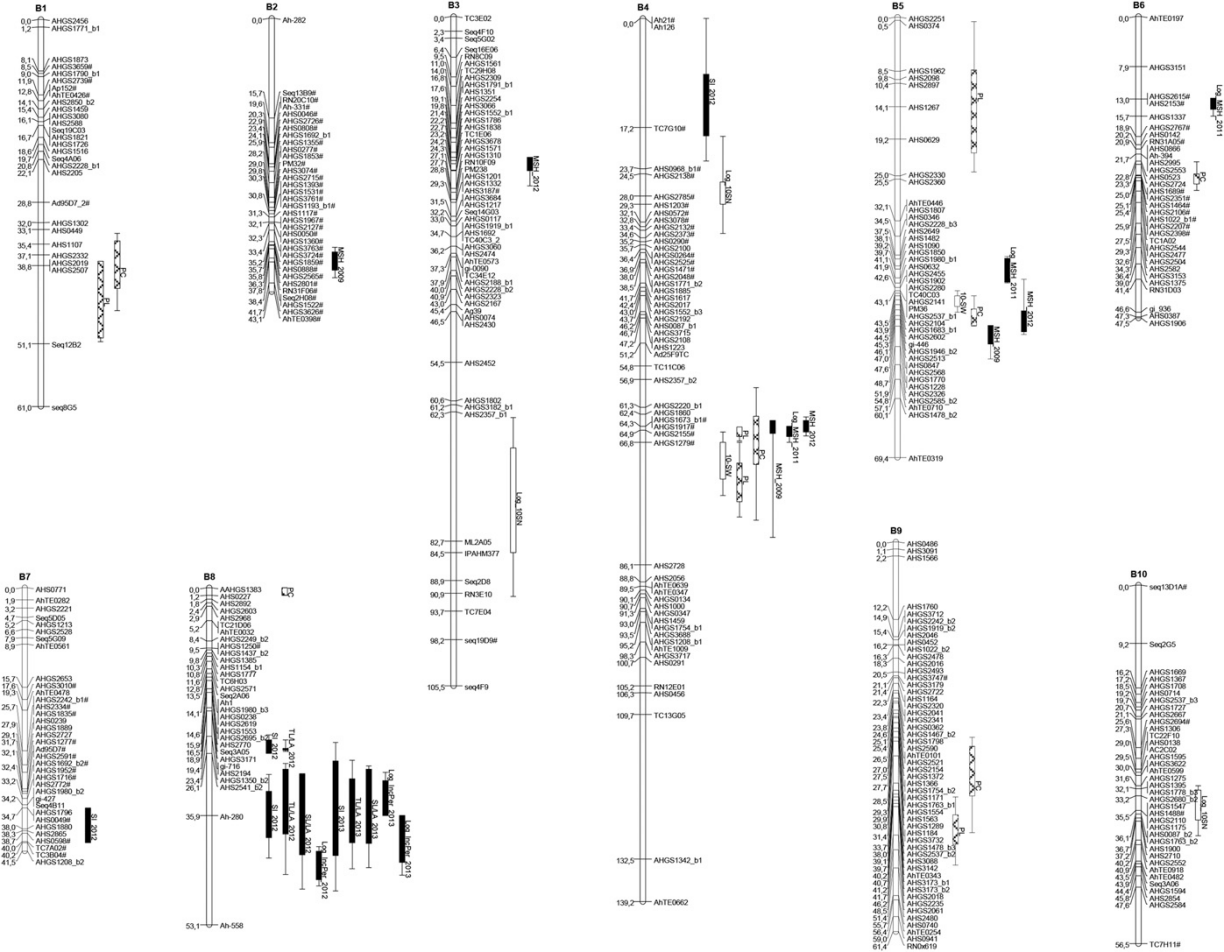
A genetic linkage map of the B-genome of *Arachis* obtained through the analysis of 94 F_6_ plants, generated from a cross between *A. ipaënsis* K 30076 and *A. magna* K 30097. Numbers on the left of each group are Kosambi map distances (cM). Markers that amplified more than one loci have numbers _1 and _2 after the marker name. Quantitative trait loci (QTL) are indicated as colored bars running alongside linkage groups. Colors/textures are according to categories: black, rust resistance [total number of lesions/leaf area (TL/LA), number of sporulated lesions/leaf area (SL/LA), susceptibility index (SI), and Incubation Period (IncPer)]; white, seed characteristics [seed number (SN) and 10-seed weight (10_SW)]; textured, plant architecture [main stem height (MSH)]; and domestication traits [peg length (PL) and pod constriction (PC)]. Distorted alleles (*P* < 0.05) are indicated by #.

All markers evaluated in this study were amplified by the use of heterologous primers. With the use of minimum LOD score of 6.0 and a maximum recombination fraction (θ) of 0.35, and after the exclusion of co-segregating markers, we found that 399 markers mapped onto 10 LGs, spanning a total map distance of 678.2 cM. These markers included 378 microsatellites and 21 transposon (miniature inverted-repeat transposable element) markers. LGs were numbered and oriented essentially according to Moretzsohn *et al.* (2009), but with B05 and B08 reoriented (“flipped”) to ensure forward compatibility with pseudomolecules produced by the Peanut Genome Sequencing Consortium (www.peanutbase.org; Genbank GCA_000816755.1). LGs ranged from 41.5 cM (with 35 markers) to 139.2 cM (55 markers), with an average distance of 1.7 cM between adjacent markers. A total of 91 (22.8%) of the 399 mapped markers deviated from the expected 1:1 ratio at *P* < 0.05 level. Of these, 79 markers were skewed toward *A. ipaënsis* and only 12 markers toward *A. magna*. Most LGs have few distorted markers, and LG B05 and B08 had no distorted markers. In contrast, LG B02 and the upper portion of LG B04 were almost entirely composed of distorted markers. These two LGs and LG B06 were distorted toward *A. ipaënsis* alleles. Distorted markers at *P* < 0.05 were identified by # (Figure 3).

#### QTL identification:

The framework map, containing 399 markers, was used for QTL analysis. LOD significance threshold estimated for each trait ranged from 2.5 to 3.2, and only QTL with LOD values exceeding these values were included. At least one QTL was detected for each of the 15 traits analyzed, with a total of 38 QTL mapped. A summary of QTL is provided in Table 1. Detailed information about markers and QTL are presented in File S1.

#### QTL for rust resistance:

**Figure 4:**
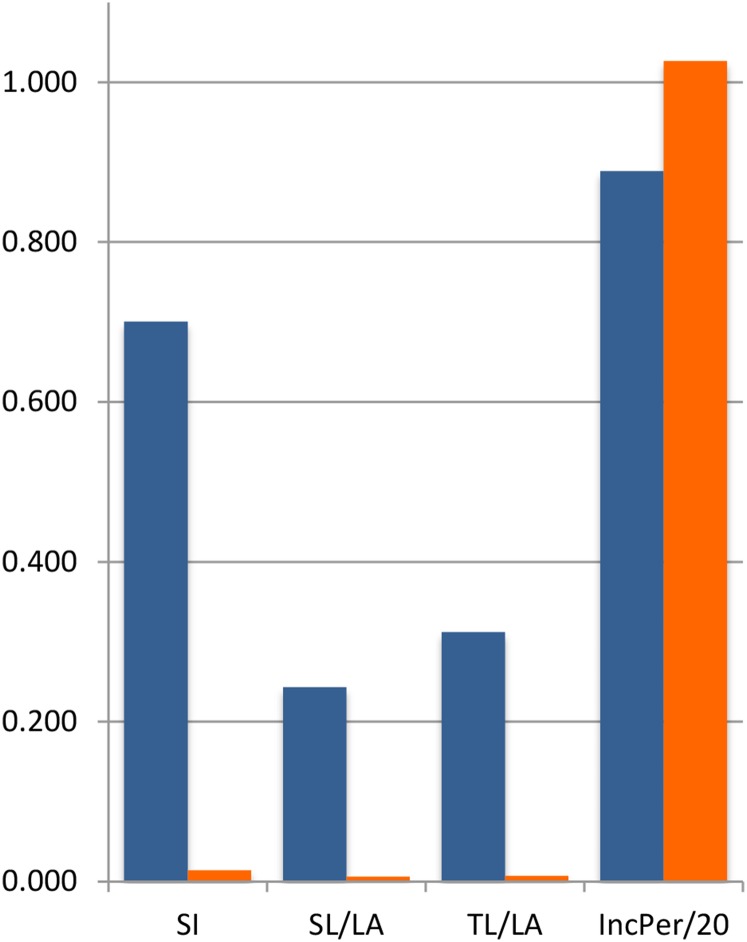
Bar graph of contribution of *A. ipaënsis* K 30076 (blue bars) *vs. A. magna* K 30097 (orange bars) alleles to the rust resistance-related traits: susceptibility index (SI), number of sporulated lesions/leaf area (SL/LA), total number of lesions/leaf area (TL/LA). and incubation period (days/20) (IncPer/20).

For rust resistance, 13 QTLs were identified on the two bioassays. A major QTL for the four components of rust resistance (SI, TL/LA, SL/LA, and IncPer) was consistently identified in both bioassays and mapped at the same marker interval on map position 35.1−42.9 cM on LG B08, with LOD scores between 2.9 and 8.2. Its closest marker is Ah-280. This QTL explained 5.8–59.3% of the phenotypic variance of the four different traits (SI, TL/LA, SL/LA, and IncPer). Another QTL for SI and TL/LA evaluated in 2012 and for IncPer_2013 was found at 25.4−33.1 cM on the same LG B08 (Table 1, Figure 3), explaining 13.2–34.8% of the total variance in 2012 and 2013, respectively. The closest markers are AHGS1350 and AHGS2541. In addition to these two QTL, two minor QTL were identified in LGs B04 and B07 for SI_2012. For all QTL, resistance was derived from *A. magna*. The contribution of *A. magna* alleles for the rust resistance-related traits was evaluated by calculating the average phenotypes of the homozygote plants for the *A. magna* allele *vs.* the average phenotype of those homozygote for the *A. ipaënsis* allele (Figure 4). *Arachis magna* alleles contributed significantly for the reduction of SI, TL/LA, and SL/LA. The effect of *A. magna* alleles was less pronounced for IncPer (Figure 4).

#### QTL for other agronomic/domestication traits:

Agronomic and domestication traits were evaluated in three different years. QTL were consistent between years; therefore, for final QTL analyses, data from different years were pooled and considered different replicates of the same experiment. The exception was MSH, which was not pooled, because measures were performed in different stages of plant growth in each year. In total, 25 QTL were identified. Interestingly, the QTL for two domestication traits (PC and PL) were placed in the same map positions: 37.1−40.8 cM in LG B01 and 64.3−64.9 cM in LG B04 (Table 1, Figure 3). LG B04 also harbors a cluster of QTL for MSH at 64.3−64.9 cM, explaining 10.7–30.4% of the total phenotypic variance. For all these traits, the main markers associated were AHGS1917 and AHGS2155. Alleles increasing values of domestication-related traits originated from both parents. However, alleles increasing seed number are all derived from *A. ipaënsis*. No QTL for domestication/productivity traits co-localized with the rust resistance QTL.

### KASP primer design and validation on a tetraploid background

**Figure 5:**
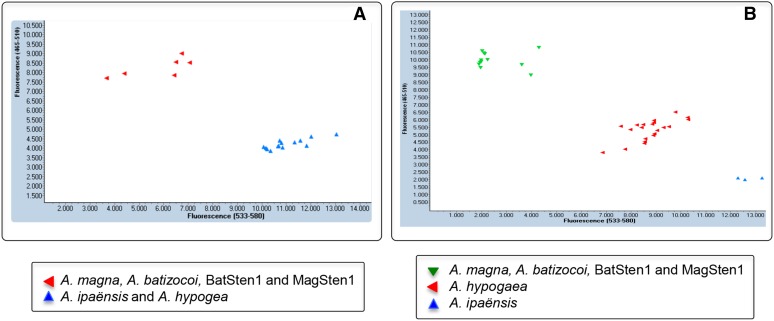
Screenshots of the two most common examples of *Arachis* B-genome single-nucleotide polymorphism genotyping using Kompetitive allele-specific polymerase chain reaction assays. Both patterns show differentiation between *A. ipaënsis* K 30076 and the B-genome of *A. hypogaea* from the wild species *A. magna* K 30097 and *A. batizocoi* K 9484 and the induced allotetraploids MagSten and BatSten1. In (A), two clusters are present: one with *A. ipaënsis* and all *A. hypogaea* cultivars, and another with the wild species and induced allotetraploids (noted in File S1 as Ah = Ai≠(Ab = Am = MagSten = BatSten)). In (B), three clusters are present. In these cases, *A. hypogaea* forms a different cluster, intermediate in position [noted in File S1 as Ah≠Ai≠(Ab = Am = MagSten = BatSten)]. All genotypes with *A. batizocoi* derived rust-resistant cluster in different groups to the susceptible genotypes.

Of a total of 24 assays designed, 22 worked well with the samples tested. Nineteen of them successfully distinguished *A. ipaënsis* and *A. hypogaea* from *A. magna* and *A. batizocoi*. Four cluster configurations were observed: (1) with nine assays, two clusters were present: one with *A. ipaënsis* plus the six cultivars of *A. hypogaea* and another with *A. magna*, *A. batizocoi*, and their derived induced allotetraploids MagSten and BatSten1 (noted in File S1 as Ah = Ai≠(Ab = Am = MagSten = BatSten)). An example is shown in Figure 5A; (2) with eight assays, one extra cluster was observed: *A. hypogaea* formed a different cluster intermediate in position (noted in File S1 as Ah≠Ai≠(Ab = Am = MagSten = BatSten))(Figure 5B); (3) with two assays, a different extra cluster was observed: MagSten was distinguished from all other genotypes (noted as (Ah = Ai)≠(Ab = Am = BatSten)≠MagSten)); (4) and finally, in three assays, *A. ipaënsis* formed an isolated cluster, being the other cluster formed by all the other genotypes (noted as Ai≠(Ah = Ab = Am = MagSten = BatSten)).

## Discussion

In this study, we aimed to generate the information and tools for the introgression of genomic regions of the diploid *A. magna* K 30097 that confer rust resistance into allotetraploid cultivated peanut, the information being the definition of the QTL that control disease resistance, and the tools being genetic markers to track the genomic regions of interest in a tetraploid genetic context (the context of introgression).

For QTL mapping, we used a progeny derived from a cross between the most likely B genome ancestor of cultivated peanut *A. ipaënsis* K 30076 (susceptible to rust), and the wild species *A. magna* K 30097 (resistant to rust). *Arachis ipaënsis* is very closely related to the B genome of cultivated peanut and has only ever been identified and collected from a single site in the wild. *Arachis magna* is very closely related to it (Krapovickas and Gregory 2007; Bechara *et al.* 2010; Moretzsohn *et al.* 2013) and may even be considered the same biological species (J. Valls, personal communication). We have used the progeny from this cross in two previously published studies. In the first, an F_2_ progeny was used to produce a map with 149 markers in 10 LGs, spanning a total of 1294 cM (Moretzsohn *et al.* 2009). In the second, a higher density genetic map of 798 loci, but covering only 461 cM, was constructed using F_6_ RILs generated by single seed descent. This map was integrated into a consensus framework of the separate diploid A and B genome components and tetraploid peanut (Shirasawa *et al.* 2013). Here, we used the same F_6_ lines and genotyping information from some additional markers to develop a map consisting of 399 high quality markers, none of which cosegregated. This map covered 678 cM. The differences in sizes of these maps, generated from the same parents, seem to be best explained by the different type of software used and parameters that treat genotyping errors in different ways (Bertioli *et al.* 2014). The largest size (1294 cM) was obtained with Mapmaker without error detection, the intermediate size (678 cM) was obtained by Mapmaker with error detection (Lincoln *et al.* 1992), and the smallest (461 cM) was obtained with Joinmap, a program that contains its own algorithms that detect error and correct genetic distances. Although it seems clear that the F_2_ genetic map distance of 1294 cM must be substantially inflated, it is unclear which of the other two estimates is better. Nevertheless, the map presented here has markers positioned with very high confidence, and, because it contains no cosegregating markers, it is better suited to QTL detection.

The main focus of this study was the identification of disease resistance QTL. However, we also investigated QTL for domestication traits. Because *A. ipaënsis* and *A. magna* are both wild species, they differ little for these traits, both having long pegs and PCs and small seeds. However, many segregants present somewhat more cultivated-like characteristics: 38 segregants have shorter pegs, 32 shorter PC, and eight larger seeds than both parents. New allelic combinations conferred traits that were not within the range of the parents. The positioning of these QTL may be of use in localizing cryptic wild alleles that could be used to improve cultivated peanut. However, we can expect that these traits are complex, quantitative, and highly dependent on the environment. Despite this, QTL were surprisingly consistent between years. We can also expect that the effects of these QTL vary with genetic background. Unfortunately, comparison of QTL for similar phenotypes identified by Foncéka *et al.* (2012a,b) in studies of wild alleles incorporated into a cultivated genetic background is difficult because of difficulty of comparing maps. Furthermore, interpretation of the significance of possible overlaps is also problematic because of the numerous QTL identified in both studies.

For the identification of disease resistance QTL, we evaluated the RILs for four measures of rust susceptibility in two independent bioassays carried out in different years. These evaluations were based on the detached leaf technique that offers precise phenotyping in controlled conditions. We did not use field assays for three reasons: (1) rust occurrence in the field is very patchy, it varies from year to year and it is fragmented within the same field; (2) the usual predominance of either Late Leaf Spot (LLS) or Early Leaf Spot (ELS) complicates evaluation (Subrahmanyam *et al.* 1982; Sujay *et al.* 2012); and, finally, (3) the extreme difference of architecture between wild genotypes and cultivated peanut would have made the field score system meaningless for this study. We anticipate the use of field evaluations later in our research within a backcrossing scheme, when advanced lines with wild segments have similar architecture to the cultivated recurrent parental, then, the occurrence of several diseases will be evaluated concomitantly.

Here, by the use of detached leaf assays, different phenotypes of disease resistance were observed. Some progenies developed no lesions. In others, lesions appeared and then necrotic tissue developed around them in a way that resembled a hypersensitive reaction. These lesions did not sporulate. Furthermore, delayed incubation period also was observed. This finding suggests multiple resistance mechanisms and genes involved in resistance. In total, we detected 13 QTL with LOD scores above the limits established by the permutation tests (Churchill and Doerge 1994; Doerge and Churchill 1996), which ranged from 2.5 to 3.2. All alleles that confer resistance were derived from the resistant parent *A. magna*. They confer less and smaller lesions, lesions with less sporulation, and longer disease incubation period. Although multiple QTL were detected, one particular marker locus (Ah-280) located on LGB08 is linked to QTL associated to all the four components of rust resistance evaluated in both years. The differences between the average phenotypes of RILs that harbor the *A. magna* allele of this marker compared with those that harbor the *A. ipaënsis* one are striking (Figure 4). The effect of *A. magna* alleles on incubation period appear less pronounced. However, this is attributable to the fact that this can only be measured on a subset of genotypes: the minority that shows symptoms. Because resistant genotypes do not have an incubation period, it is not possible to measure with precision the allelic effect on this trait of the whole range of genotypes. This fact could also explains the high estimates of the proportion of phenotypic variance explained by the QTL detected, which tend to be inflated by the small number of progenies with that particular trait.

Because this QTL confers such strong disease resistance, and it is located at the very end of the chromosome of a species with very close affinity to the B genome of cultivated peanut, it is unlikely to suffer strong linkage drag. Overall it seems like an ideal candidate for the introgression of rust resistance into cultivated peanut. To facilitate this, we developed multiple markers surrounding the locus Ah-280. Also, markers were developed around the locus AHGS1350, which is linked to two strong QTL. To do this, we identified SNPs between *A. magna* and *A. ipaënsis* around these loci. Because of the very high similarity of *A. ipaënsis* to the B genome of *A. hypogaea*, 19 of 22 KASP markers designed successfully distinguished the rust-resistant wild genotypes and their allotetraploid derivatives from *A. ipaënsis* and *A. hypogaea*. For nine of the assays, the *A. ipaënsis* and *A. hypogaea* genotypes clustered together; for the other eight, the clusters of the *A. hypogaea* were shifted to an intermediate position because of the interfering signal generated from the DNA bases on the homeologous A-genome (as described in Bertioli *et al.* 2014). This distortion of clustering is easy to account for and does not affect the function of the markers.

Previous studies of rust resistance in pure *A. hypogaea* have yielded limited information. There are indications that rust-resistance is controlled by a few major genes (Van der Plank 1963). However, a quantitative genetic analysis of parents and progenies indicated that rust resistance is more complex and does not fit a typical race specific pattern (Subrahmanyam *et al.* 1993). More recent studies in which authors used germplasm where one of the parents has a small genomic contribution from wild species have been more informative. These have identified a major QTL for rust resistance that explains up to 83% of the phenotypic variance (Khedikar *et al.* 2010; Sujay *et al.* 2012). The origin of this resistance is almost certainly a chromosome segment derived from the A-genome species *A. cardenasii*. This segment was likely inherited from the common parent GPBD4, which in turn has a parent ICGV 86855 that is derived from an *A. hypogaea* × *A. cardenasii* cross (Gowda *et al.* 2002). Microsatellite markers on this QTL region currently are being used for marker-assisted backcrossing, with very promising results (Varshney *et al.* 2014). The QTL was located in LG AhXV according to a consensus map published for *A. hypogaea* (Gautami *et al.* 2012). Sequence similarity searches of the five markers linked to this QTL against the recently available pseudomolecule sequences of *A. duranensis*, the most probable A-genome ancestral species of cultivated peanut (www.peanutbase.org; Genbank accession PRJNA258023), indicate that best matches for four of them are at the end of chromosome 3 (Aradu.A03) between 131.3 and 133.7 Mbp (File S1, *A. cardenasii*-rust-QTL-markers). Therefore, this QTL and the one identified in this study would appear to be distinct and could, in principle, both be incorporated into the same peanut cultivar to provide stronger and more durable resistance.

For the introgression of chromosomal segments from *A. magna* K 30097, an allotetraploid with *A. stenosperma* ([*A. magna* × *A. stenosperma*]^4x^; MagSten) has been created (A. Favero, personal communication) and it is being used in the peanut breeding program in the Instituto Agronômico de Campinas, São Paulo, Brazil, in a collaborative effort with Embrapa Genetic Resources and Biotechnology, Brasília, Brazil (Leal-Bertioli *et al.* 2014a). The SSR (Ah-280) and KASP markers described here are now being used for the selection of backcrossed lines that harbor the rust QTL in this breeding program. We anticipate that this will greatly facilitate the testing of function of this chromosomal segment in the genetic background of cultivated peanut.

During the past two decades, the introgression of wild alleles for resistance to pests and diseases has proven very valuable for the peanut crop. Wild alleles confer the strongest known resistance phenotypes not only against rust (Varshney *et al.* 2014) but also late leaf spot (Khedikar *et al.* 2010) and root-knot nematode (Stalker *et al.* 2002; Simpson *et al.* 2003; Chu *et al.* 2011; Burow *et al.* 2014). These alleles have proven to be stable over different environments and in different genetic backgrounds. The use of alleles harbored on wild chromosome segments also has facilitated the use of molecular markers for backcross selection because the segments have a high rate of DNA polymorphism relative to cultivated peanut. The improved understanding of wild and cultivated species relationships (Milla *et al.* 2005; Seijo *et al.* 2007; Burow *et al.* 2009; Robledo *et al.* 2009; Moretzsohn *et al.* 2013; Leal-Bertioli *et al.* 2014b), increased ease of marker development, and better understanding of genome structure that is being gained from genome sequencing of peanut’s diploid ancestors (www.peanutbase.org) are likely to facilitate the greater use of wild alleles and enable further gains for the peanut crop.
